# Elucidation and prevention of an unexpected methylamine byproduct formed during trifluoroacetic acid-mediated side chain deprotection of azido-containing peptides

**DOI:** 10.21203/rs.3.rs-6296173/v1

**Published:** 2025-04-03

**Authors:** Yixin Xie, Tania L. Lopez-Silva, Marzena A. Dyba, Tanja Grkovic, Joel P. Schneider

## Abstract

Purpose We found that during TFA-mediated cleavage of azide-containing peptides during solid-phase peptide synthesis a byproduct characterized by a difference of 12 mass units is formed. This study identifies this byproduct, provides a rationale for its formation and a solution for its inhibition. Method NMR and HRMS analyses as well as chemical synthesis is employed to identify the byproduct and probe the mechanism of its formation. Results Data is consistent with the conversion of azide to methylamine which occurs during peptide cleavage likely via a Schmidt rearrangement involving nucleophilic azide attack of t-butyl cations generated during side chain deprotection of Boc- and t-butyl ether groups. Conclusion Installing azide-containing residues using the newly reported 2-(trimethylsilyl)ethoxycarbonyl (Teoc)-protected compound **3a** proved effective at significantly thwarting the formation of methyl amine byproduct.

### Introduction

The incorporation of azides into peptides via solid-phase synthesis is commonplace, given their utility in facilitating chemical ligation reactions. (Holub and Kirshenbaum 2010, Ahmad Fuaad, Azmi et al. 2013, Tang and Becker 2014, Medina and Schneider 2017) Fmoc-azido-lysine is a commercially available residue often used to install the azido group. However, the azido-lysine side chain is hydrophobic, and the incorporation of this residue decreases the solubility of the final peptide in aqueous solution. This effect can be dramatic when multiple copies of the azide-containing residue are incorporated within a single sequence. We recently developed the positively charged azido-containing amino acid **1** to address this issue, **Figure 1**. (Xie, Lopez-Silva et al. 2022) In addition to an azide group, this residue contains a secondary amine within its side chain, which when protonated at neutral pH, significantly increases the residue's hydrophilicity. Typically, incorporation of azide residues involves standard Fmoc-based solid phase peptide synthesis (SPPS) protocols employing commonly used coupling reagents and cleavage cocktails affording crude material mainly comprised of the desired azido-peptide.

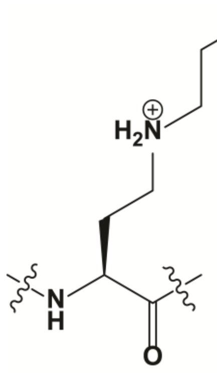

**1**

**Figure 1**. Residue 1

However, over the course of utilizing residue **1** in the synthesis of a diverse set of peptides, we observed the formation of an unknown byproduct. If only a single azide-containing residue is incorporated into a sequence, the amount of byproduct formed is minimal, allowing purification of the desired peptide. However, the incorporation of multiple azido residues led to the formation of substantial amounts of undesired material, which became problematic. To elucidate the identity and genesis of this byproduct, we re-examined mass spectrometry data from previous synthetic campaigns where residue **1** and commercially available azido lysine were used. Interestingly, independent of whether azido lysine or residue **1** was incorporated, byproduct formation occurred. This was surprising since, to our knowledge, there have been no reports of side reactions involving the commercially available and extensively used azido lysine residue. Further, it suggested that byproduct formation was not dependent on the exact residue but on the inclusion of the azide functional group.

Examining mass spectrometry data across all the peptides prepared in the lab containing either azido lysine or residue **1** indicated that byproduct formation was dependent on the amino acid composition of the peptide, and that the molecular weight of the byproduct was always 12 mass units smaller than the desired peptide. Herein, we use a combination of chemical synthesis, high-resolution mass spectrometry (HRMS) and nuclear magnetic resonance (NMR) analysis to identify the byproduct, propose a rationale for its formation, and provide a means to overcome its formation. As will be shown, byproduct formation does not occur during peptide elongation on the resin but rather, during the final cleavage and side chain deprotection step. Specifically, side chain azido groups react with t-butyl cations generated during trifluoroacetic acid (TFA)-mediated side chain deprotection, converting the azide into a methyl amine. Byproduct formation can be overcome by installing the azide group via the newly reported compound **3a**, which contains a 2-(trimethylsilyl)ethoxycarbonyl (Teoc) side chain protecting group that greatly limits this side reaction, **Figure 2**.

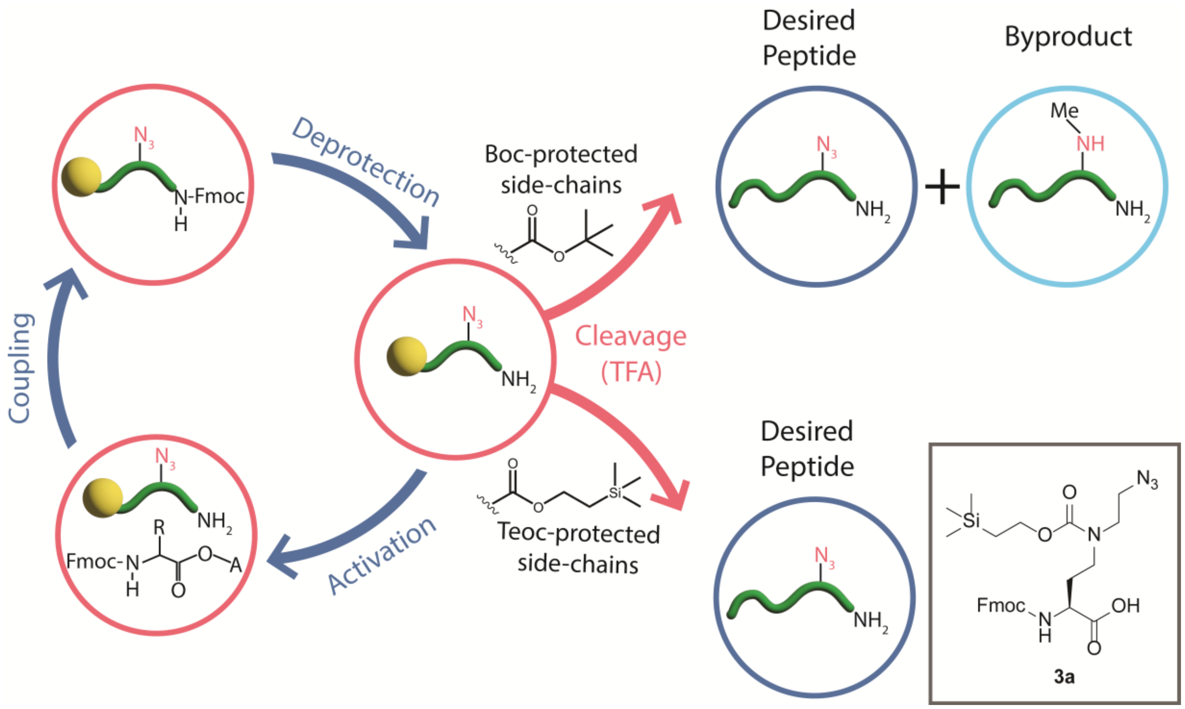

**Figure 2**. Methylamine byproduct formed during TFA-mediated side chain deprotection of azido-containing peptides. Byproduct formation results from the reaction of azide with t-butyl cations generated during the peptide cleavage/side chain deprotection step. Byproduct formation is limited by employing Teoc-protected residue **3a**.

### Materials and Methods

All reactions were conducted in flame dried round-bottom flasks. Automated column chromatography was performed on a Teledyne ISCO Combiflash Rf using RediSep Rf Gold® C18 Reversed Phase Columns (20-40 µm). Commercially available chemicals and solvents were purchased from Sigma-Aldrich, Ambeed, Fisher Scientific, TCI Chemicals, AA blocks and AK scientific. All reagents and solvents were used as received without additional treatment or purification unless specified. Deuterated solvents were purchased from Sigma-Aldrich. Due to the potential explosive nature of azido-containing compounds, careful handling is essential.

### Peptide Synthesis

Fmoc solid-phase peptide synthesis with ProTide Rink amide resin was performed at 0.1 mmol scale. Tripeptides and tetrapeptides were synthesized manually. Fmoc-protected amino acids (0.5 mmol, 5 eq, 0.2 M in DMF) were pre-activated with HATU (0.5 mmol, 5 eq, 0.2 M in DMF) and DIPEA (0.5 mmol, 5 eq, 0.3 M in DMF) for 5 min. (Carpino, Imazumi et al. 2002) The coupling solution was added to the resin and shaken for 45 min. Coupling completion was assessed by the Kaiser Test. The resin was washed with DMF (3X) and DCM (3X) before the next cycle. Fmoc deprotections were carried out with 20% piperidine in DMF at room temperature twice per cycle (10 mL, 5 min). After each deprotection, the resin was washed with DMF (5X). Once the peptide sequence was completed, the resin was transferred to a fritted vessel and washed with DCM (3X), DMF (3X), and DCM (2X). Then, the resin was dried in a vacuum manifold for 30 min.

Other peptides were synthesized on a CEM Liberty Blue™ microwave-assisted peptide synthesizer. Couplings for all amino acids unless stated otherwise were performed with Fmoc-protected amino acids (5 eq, 0.2 M in DMF), Oxyma (5 eq, 1 M in DMF), and DIC solution (5 eq, 0.5 M in DMF).(Subirós-Funosas, Prohens et al. 2009) The reaction was heated at 90°C for 4 min. For the peptide VXVXVXVXVpPTXVEVXVXV, the valine residue immediately following the D-proline was triple coupled at room temperature with HCTU (5 eq, 0.45 M in DMF) and DIPEA (10 eq, 2 M in NMP) for 30 min. The subsequent two residues were double coupled using the same HCTU and DIPEA conditions.

In general, peptides were cleaved from the dried resin with 10 mL of a cleavage cocktail comprised of 95% TFA, 2.5% triisopropylsilane, and 2.5% water. Other cleavage conditions screened for peptide XIA and $^{N3}$KVSA are listed in table 1. After stirring at room temperature for 3 h, TFA was removed by a stream of argon gas at room temperature. Crude peptides were precipitated with cold diethyl ether, collected by filtration, washed with diethyl ether, dissolved in water, and lyophilized before purification. All peptides contain free amino N- and amidated C-termini.

### High performance Liquid Chromatography (HPLC) Analysis

HPLC was performed using a solvent system of 0.1% TFA in Milli-Q $H_2O$ (Standard A) and 0.1% TFA in 9:1 $CH_3CN/H_2O$ (Standard B). Analytical RP-HPLC was performed on an Agilent 1200 series HPLC, using a Vydac 218TP C18 column (250 x 4.6 mm, 5 µm), with a linear gradient from 0 to 100% B at a rate of 1% B per min, at a flow rate of 0.25 mL/min and a column temperature of 40°C. Preparative RP-HPLC was performed on a Waters 600 system, equipped with a Waters 2489 UV detector and a Vydac 218TP C18 column (250 x 22 mm, 10 µm), at a flow rate of 8.0 mL/min and a column temperature of 40°C. Analytical HPLC chromatograms of purified peptides and associated mass spectroscopy can be found in the supporting information of reference. (Xie, Lopez-Silva et al. 2022)

### Liquid Chromatography Mass Spectrometry (LCMS)

Analytical LCMS was performed on a Shimadzu LCMS 2020, using a Luna C18 column 100 Å (150 x 3 mm, 5 µm), with a linear gradient from 0 to 90% B at a rate of 2% B per min and a column temperature of 40°C. Solvents for LCMS consisted of 0.1% formic acid in $H_2O$ (Standard A) and 0.1% formic acid in $CH_3CN$ (Standard B).

### Nuclear Magnetic Resonance (NMR)

NMR was performed on a Bruker AV400 spectrometer. Chemical shifts (d) are reported in parts per million (ppm) relative to the solvent signal. Spin-spin coupling constants, J, are reported in Hz. Multiplicities are reported as follows: singlet (s), doublet (d), triplet (t), quartet (q), multiplet (m). Structural assignments were made with additional information from the HSQC and HMBC experiments.

### Tandem High Resolution Mass Spectrometry

Mass spectrometry data were acquired on an Agilent 6520 Accurate-Mass Q-TOF LC/MS/MS System, (Agilent Technologies, Inc., Santa Clara, CA) equipped with a dual electro-spray source, operated in the positive-ion mode. Separation of peptides was performed on a Poroshell 300SB-C18 column (2.1 mm x 75 mm; particle size 5 μm). In full scan and MS/MS experiments, the analytes were eluted at a flow rate of 1 mL/min with a 5 to 100% organic gradient over 4 minutes, followed by isocratic conditions at 100 % organic phase for 1 minute. Both mobile phases, water, and acetonitrile, contained 0.1% formic acid. The instrument was used in a full-scan TOF mode or product ion scan (MS/MS) mode. MS source parameters were set with a capillary voltage of 3.5 kV, a fragmentor voltage of 180 V and skimmer of 65 V. The gas temperature was 350 °C, drying gas flow was 12 l/min and nebulizer pressure was 55 psig. Nitrogen was used as a collision gas. Data were acquired at high resolution (3,200 *m/z*) mode at frequency 4 GHz. TOF-MS mass spectra were recorded across the range 100–3,200 m/z. Q-TOF-MS/MS experiments were carried out in the range 100–3,200 *m/z* with a scan rate of 4 spectra/s and they were accomplished with collision energy of 50 V.

To maintain mass accuracy during the run time, an internal mass calibration sample was infused continuously during the LC/MS runs. Data acquisition was performed using Mass Hunter Workstation (version B.06.01). For data analysis of mass spectra, the Mass Hunter Qualitative Analysis software (version B.07.00) with Bioconfirm Workflow was used. The software provides resolved isotope deconvolution algorithm to calculate peptides' molecular weights and formula generation for peptide identification. For identification of fragment ions from MS/MS experiments online Proteomics Toolkit, a fragment ion calculator was used.

To determine the regiospecific nature of azide to methylamine conversion with respect to the peptide containing multiple azide functionalities (VXVXVXVXVpPTXVEVXVXV, where X = residue **1**), targeted MS/MS fragmentation by collision-induced dissociation was used. Singly and doubly charged *b*- and *y*-ion series were observed on the product-ion spectra of the Δ12 byproduct.

### Results and Discussion

Over the last several years we had prepared a family of peptides containing either azido lysine ($^{N3}$K) or azido-containing residue **1** to be used in 1,3-dipolar cycloaddition (Click) reactions, **Figure 3A**. We employed Nα-Fmoc-, and Nα-Fmoc-/δ-Boc-protected analogs of these residues, respectively, in standard solid phase peptide synthesis. Close examination of nominal resolution mass spectrometry data of crude peptide (before HPLC

A)

| Peptide sequence | Expected azido-peptide mass | Byproduct mass | Δ |
|---|---|---|---|
| **XIA** | 371.2 | 359.3 | 12 |
| **FSXV** | 520.2 | 508.4 | 12 |
| **YGGASVXA** | 792.4 | 780.5 | 12 |
| **FQXV** | 561.3 | 549.5 | 12 |
| **FRXV** | 589.4 | 577.5 | 12 |
| **FHXV** | 570.3 | 558.5 | 12 |
| **$^{N3}$KVSA** | 429.2 | 417.4 | 12 |
| **YGGASV$^{N3}$KA** | 777.4 | 765.5 | 12 |
| **VXVXVXVXVpPTXVEVXVXV** | 2517.5 | 2505.6<br>2493.7<br>2481.8 | 12<br>24<br>36 |
| **$^{N3}$KIA** | 356.2 | N/A | N/A |

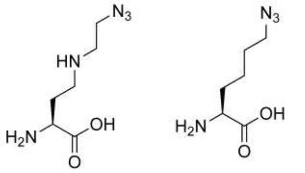

**1 = X** **$^{N3}$K**

B)

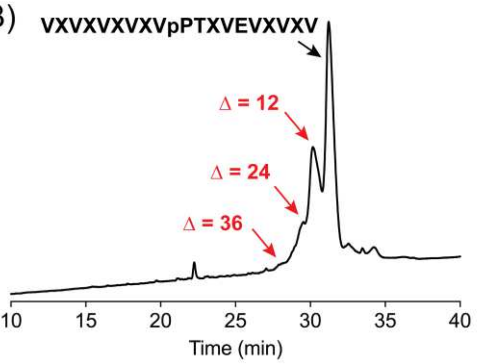

**Figure 3**. (A) Peptide sequences containing azido side chains used in this study along with their calculated mass $(M+H)^+$, observed byproduct mass $(M+H)^+$, and mass differences. N/A = not observed. All peptides contain free amino N- and amidated C-termini. (B) HPLC chromatogram of the crude peptide VXVXVXVXVpPTXVEVXVXV and mass differences (relative to desired peptide) corresponding to the multiple byproducts present in the sample.

purification) showed the formation of a minor byproduct characterized by a molecular weight 12 mass units smaller than the desired peptide mass. When multiple copies of residue **1** were incorporated into a sequence, for example (VXVXVXVXVpPTXVEVXVXV, where X = **1**), multiple byproducts were observed, each having a molecular weight that was a multiple of D12 mass units, **Figure 3B**. In this case, the most abundant byproduct (Δ = 12) comprised over 30% of the crude material. The fact that the abundance of the byproduct increased with increasing number of azido groups present in a given peptide suggested that byproduct formation involved azide-based chemistry. One possibility involves known conversions of the azide to amine by chemical reduction (Scriven and Turnbull 1988, Schneggenburger, Worbs et al. 2010) or photolysis (Low, Kang et al. 2004, Abbenante, Le et al. 2007), but neither reaction is consistent with our synthetic protocol and primary amine formation is not consistent with the mass spectrometry data.

We employed the small tripeptide (XIA, where X = residue **1**) for our initial studies to identify the structure of the byproduct by HRMS. The peptide was synthesized, cleaved from resin and the byproduct isolated by preparative HPLC. HRMS provided a parent ion having an associated mass of 359.2767 Da, which suggests a molecular formula of $C_{16}H_{35}N_6O_3$. The mass error between the observed and calculated mass is 0.37 ppm. This chemical formula corresponds to the loss of two nitrogen atoms and a gain of one carbon atom and four hydrogens compared to the parent azido peptide XIA. This suggests a conversion of the azido group to a methylamine functional group through loss of $N_2$. The masses of two fragment ions, originating from the cleavage of the parent byproduct's two amide bonds, provide chemical formulas that are also consistent with this chemical transformation, **Figure 4A**.

$^1$H NMR of the isolated byproduct is also consistent with methylamine formation, providing a spectrum that can be assigned to the modified peptide ($^{Me}$XIA, see structure in **Figures**

**4A** and **D**). The newly formed methyl group provides a sharp singlet at 2.77 ppm that integrates for three protons, **Figure S1**. An HSQC experiment shows correlation between the methyl protons (H1 at 2.77 ppm) and the carbon (C1 at 32.5 ppm), confirming the presence of the methyl group, **Figure 4B**. The fully assigned HSQC spectrum can be found in **Figure S2**. Further, an HMBC experiment showed correlation of H1 at 2.77 ppm to C2 at 46.1 ppm. This three-bond coupling defines the connectivity of the methyl group to the $\varepsilon$-carbon of the residue side chain, **Figure 4C**. Unfortunately, due to the limited solubility and low concentration of the byproduct, additional side chain correlations were difficult to observe. Lastly, the structure of $^{Me}$XIA was verified by preparing the methylamine-containing peptide *de novo* using compound **3c**, **Figure 7**. The $^{1}$H NMR of synthesized $^{Me}$XIA was collected and compared to the spectrum of byproduct $^{Me}$XIA isolated from the synthesis of XIA (where X = residue **1**). **Figure 4D** shows that the spectra are nearly identical, with the spectrum of the isolated byproduct showing a small degree of line broadening in the amide/amine region.

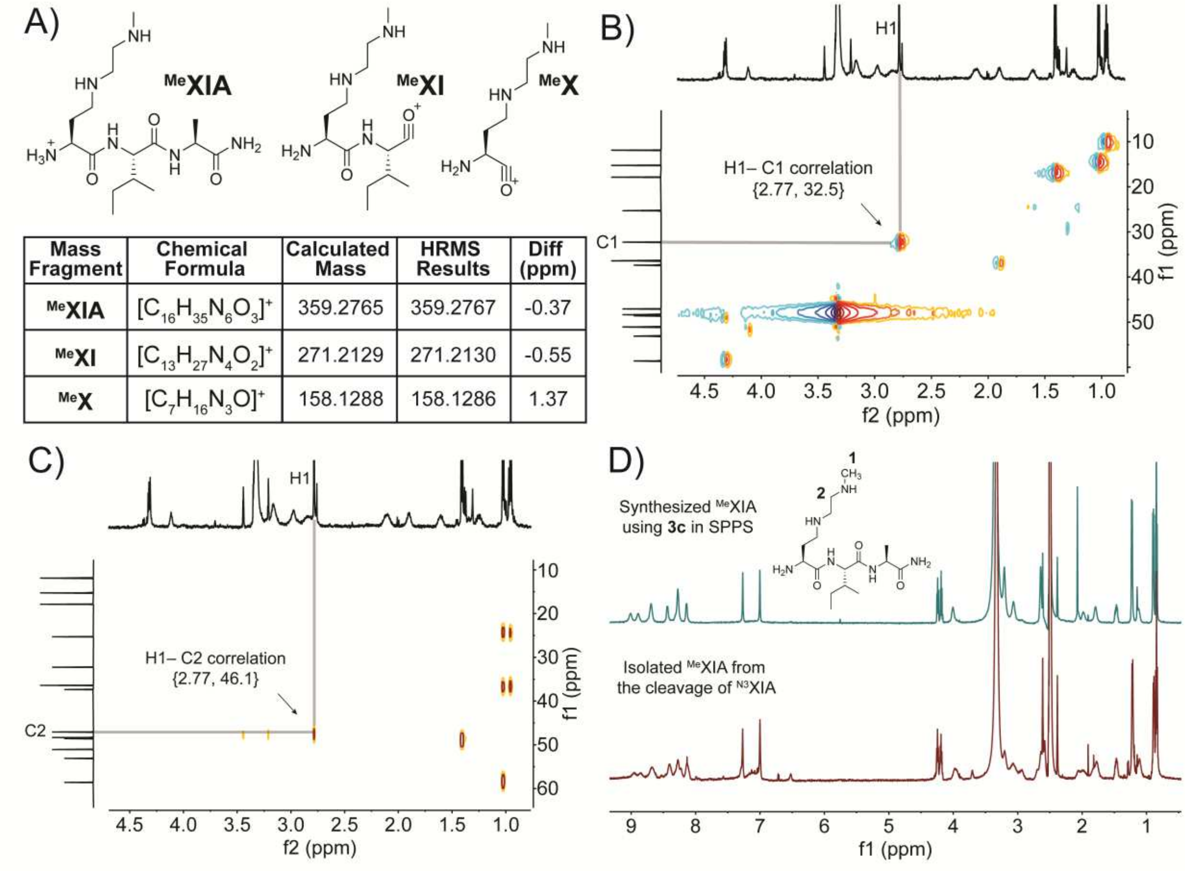

| Mass Fragment | Chemical Formula | Calculated Mass | HRMS Results | Diff (ppm) |
|---|---|---|---|---|
| $^{Me}$**XIA** | $[C_{16}H_{35}N_6O_3]^+$ | 359.2765 | 359.2767 | -0.37 |
| $^{Me}$**XI** | $[C_{13}H_{27}N_4O_2]^+$ | 271.2129 | 271.2130 | -0.55 |
| $^{Me}$**X** | $[C_7H_{16}N_3O]^+$ | 158.1288 | 158.1286 | 1.37 |

**Figure 4.** (A) Structures, chemical formulas, calculated and observed masses of isolated byproduct $^{Me}$XIA and its mass fragmentations. Differentials of calculated versus observed masses are also provided. (B) HSQC NMR of isolated $^{Me}$XIA in Methanol-d$_4$. Arrow highlights the correlation of H1 and C1 within the methyl group. (C) HMBC NMR of isolated $^{Me}$XIA in Methanol-d$_4$. Arrow highlights the correlation of H1 and the adjacent C2. (D) $^{1}$H NMR of synthesized $^{Me}$XIA using residue **3c** and $^{Me}$XIA isolated from the chemical synthesis of XIA. Spectra collected in DMSO-d$_6$.

All of the peptides studied contain a single azido group except the peptide VXVXVXVXVpPTXVEVXVXV (where X = residue **1**), which contains a total of seven azido groups that can potentially undergo conversion to methylamine. Interestingly, only three azido groups are modified to any detectable level as shown in the chromatogram in Figure 3B. Further, the main byproduct, which comprises around 30% of the crude peptide material, contains only one modified azido group. We investigated the regiospecific nature of methylamine formation for this singly modified byproduct using tandem high-resolution mass spectrometry on the isolated byproduct. Data show that methylamine formation occurs primarily from the reaction of the most N-terminal azido group (position 2, Figure 5A). The data highlight a series of b-ions, including $b_2^+$, $b_4^+$, $b_6^+$, and $b_8^+$, corresponding to fragmentations along the peptide backbone, with the $y_{20}{}^{2+}$ ion also labeled. Modification at position 2 is defined by the $b_2^+$ ion, which is characterized by a mass-to-charge (*m/z*) ratio of 257.19714. This mass provides a calculated molecular formula of $C_{12}H_{25}N_4O_2$, indicating methylamine modification at this position, **Figure 5B**. The masses associated with $b_4^+$, $b_6^+$, and $b_8^+$ ions are also consistent with modification at position 2. Additional peaks are observed in **Figure 5A** which originate from the doubly modified byproduct, which was not entirely removed during the isolation of the main byproduct by HPLC purification. Lastly, the isotope patterning of the $b_2^+$ ion provides a 1.94 ppm difference between calculated and observed masses, further supporting the proposed molecular formula of this ion. The fact that azido modification occurs predominately near the terminus of the peptide suggests that the mechanism defining its formation may be influenced by steric hindrance, perhaps involving an intermolecular reaction as opposed to a strictly intramolecular process.

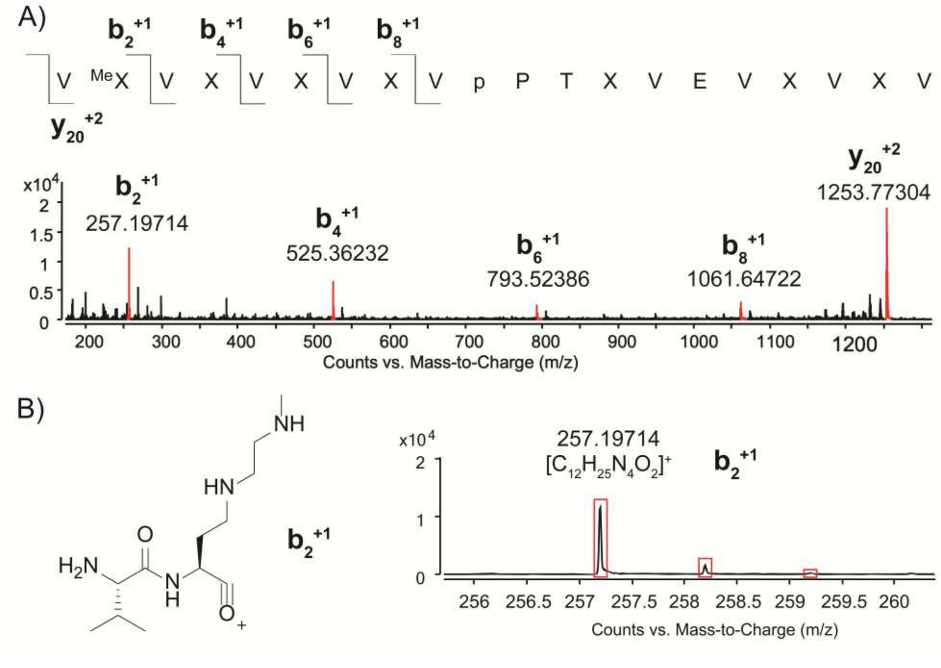

**Figure 5**. Tandem high-resolution mass spectrometry analysis of peptide $V^{Me}$XVXVXVXVpPTXVEVXVXV. (A) A subset of fragmentation ions is highlighted to show that methylamine formation occurs predominantly at position 2. (B) Structure of the $b_2^+$ ion. Isotope patterning of the $b_2^+$ ion. Peptide contains free amino N- and amidated C-terminus.

Examination of the literature provided a proposed mechanism for the formation of the methylamine byproduct, namely a Schmidt reaction involving the nucleophilic attack of azide on an electrophile that would ultimately afford the methyl group. (Grecian and Aubé 2010) The identity of the electrophile was initially unknown, but **Figure 3A** provided a clue to its identity. The only peptide sequence whose synthesis did not produce the byproduct ($^{N3}$KIA) is void of any side chain protecting groups that could produce a t-butyl cation during TFA-mediated side chain deprotection. All the other sequences listed contained either Boc- and/or t-butyl side chain protecting groups. **Figure 6A** shows a proposed mechanism that involves attack of t-butyl cation by azide, subsequent methyl migration, release of molecular nitrogen, and formation of the corresponding iminium ion. Hydrolysis of the iminium ion results in the formation of methylamine and acetone. Similar types of Schmidt rearrangements have been used in natural product synthesis. (Frankowski, Golden et al. 2008) Due to the short half-life of the intermediates and strong acidic conditions of the reaction medium, it was difficult to directly investigate this proposed mechanism by spectroscopy or spectrometry. However, we investigated the mechanism indirectly through chemical synthesis where byproduct formation was monitored by LCMS for several azido lysine-containing peptides as a function of protecting group identity and reaction conditions. No methylamine byproduct was detected in $^{N3}$KIA that lacked a protecting group in the side chain, **Figure 6B**. Further, no modification was observed in $^{N3}$KVQA prepared using trityl protected glutamine, **Figure 6C.** It is likely that the rate of the peptide-bound azide attack on the bulky trityl cation produced during TFA treatment is slower than scavengers (TIPS, $H_2O$). However, byproduct formation is observed for peptide $^{N3}$KVSA, which contained a t-butyl protected serine, **Figure 6D**. Finally, **Figure 6E** shows that when 50 equivalents of tert-butyl acetate (t-BuOAc) is deliberately added to the cleavage cocktail during the synthesis of peptide $^{N3}$KVQA, a substantial amount of the byproduct is clearly formed. This is due to the *in situ* generation of t-butyl cation from tBuOAc under acidic conditions. (Anxionnat, Guerinot et al. 2009, Naredla and Klumpp 2013) This peptide ($^{N3}$KVQA) normally does not undergo byproduct formation as shown in Figure 6C. Taken together, the data is consistent with the proposed mechanism, which occurs during the TFA-mediated cleavage and side chain deprotection step of peptide synthesis.

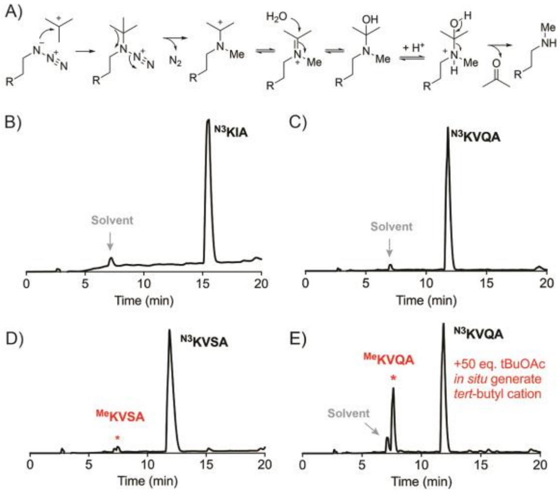

**Figure 6**. (A) Proposed Schmidt rearrangement leading to methylamine formation during TFA-mediated peptide cleavage and side chain deprotection. (B) No byproduct is formed for peptide $^{N3}$KIA, which lacks t-butyl protecting groups. (C) No byproduct is formed for peptide $^{N3}$KVQA, which contained a trityl protecting group. (D) Methylamine byproduct ($^{Me}$KVSA) is formed during the cleavage of t-butyl protected $^{N3}$KVSA. (E) Methylamine byproduct ($^{Me}$KVQA) is formed when 50 eq. of tBuOAc is added to the TFA/TIPS/$H_2O$ cleavage cocktail during cleavage of peptide $^{N3}$KVQA. All peptides contain free amino N- and amidated C-termini.

Having determined that byproduct formation was taking place during the cleavage/side chain deprotection step, we screened cleavage conditions in an attempt to prevent this undesired reaction. However, methylamine formation persisted at levels ranging from 4 - 6% in crude isolates under all conditions tested, **Table 1**. For example, we first investigated different ratios of TFA, TIPS, and $H_2O$ to optimize quenching of the t-butyl cation (entries 1-3). Surprisingly, increasing the TIPS concentration from 2.5% to 10% did not significantly reduce methylamine formation (entry 3). Alterations in the order of reagent addition were also tested. In entry 4, TIPS and water were added first to soak the resin for 10 minutes, followed by TFA, to ensure the azido group was surrounded by cation scavengers. Alternant scavengers were used, for example, increasing concentrations of triethylsilane (TES) (entries 7-9). Phenylsilane, trimethoxysilane, m-cresol were also ineffective as was reagent R consisting of TFA/anisole/thioanisole without EDT (entries 10-13). Finding reaction conditions to limit this side reaction seemed unlikely and another strategy would need to be explored.

**Table 1** Precent of by-product $^{Me}$XIA and $^{Me}$KVSA during the cleavage of $^{N3}$XIA and $^{N3}$KVSA under different cleavage conditions

| Entry | Conditions[a] | $^{Me}$XIA[b] | $^{Me}$KVSA[b] |
|---|---|---|---|
| **1** | TFA/TIPS/$H_2O$ 95/2.5/2.5 | 5.8% | 4.7% |
| **2** | TFA/TIPS/$H_2O$ 90/5/5 | 4.5% | ND |
| **3** | TFA/TIPS/$H_2O$ 80/10/10 | 4.4% | ND |
| **4** | TFA/$H_2O$ 95/5, 2h | 4.2% | ND |
| **5** | TIPS 5% $H_2O$ 5% 15min, then TFA 90% | 4.3% | 6.3% |
| **6** | TFA 95% 1H, then $H_2O$ 5% 1H | 6.8% | 5.8% |
| **7** | TFA/TES/$H_2O$ 95/2.5/2.5 | 6.0% | ND |
| **8** | TFA/TES/$H_2O$ 90/5/5, 2h | 5.2% | ND |
| **9** | TFA/TES/$H_2O$ 80/10/10, 2h | 4.6% | ND |
| **10** | TFA/ phenylsilane/$H_2O$ 95/2.5/2.5 | 4.1% | 5.5% |
| **11** | TFA/trimethoxysilane/$H_2O$ 95/2.5/2.5 | 4.3% | 5.2% |
| **12** | TFA/anisole/thioanisole 95/5/5 (Reagent R without EDT) | 6.0% | 4.7% |
| **13** | TFA/m-cresol 95/5 | 4.4% | 5.3% |

[a] Reaction scale: 5 mg resin after SPPS, 50 μL cleavage reagent
[b] Percentage calculated by LCMS of crude peptide after cleavage at 195 nm wavelength

We hypothesized that t-butyl cations generated spatially close to the azide during TFA-treatment were especially problematic, such as would be the case when compound **3b** was used to install the azide, **Figure 7**. As such, a strategy to limit byproduct formation is replacing the Boc-protecting group of **3b** with one that limits the formation of reactive electrophile during its deprotection. The 2-(trimethylsilyl)ethoxycarbonyl (Teoc) side chain protecting group should undergo acid-promoted deprotection in TIPS/$H_2O$ to produce ethylene, $CO_2$, and a silicon adduct resulting from direct nucleophilic attack of either water or hydride. (Carpino, Tsao et al. 1978) Compound **3a**, containing a Teoc-protected side chain amine, was prepared by the facile 2-step procedure outlined in Figure **7B**. The same general procedure can be used to prepare compounds **3b** and **3c**. Briefly, ozonolysis of commercially available allylglycine gives aldehyde **4** after reductive workup with thiourea in quantitative yield without further purification. The second step is a one-pot reaction involving reductive amination of **4** with amine **5** under mild acidic conditions and protection with Boc- or Teoc-protecting groups. Methanol and dioxane were used as the optimized solvents for amino acids with Boc- and Teoc-protecting group, respectively. Final yields are between 50-60% with only one flash chromatography purification, providing suitably protected residues for use in Fmoc-based SPPS.

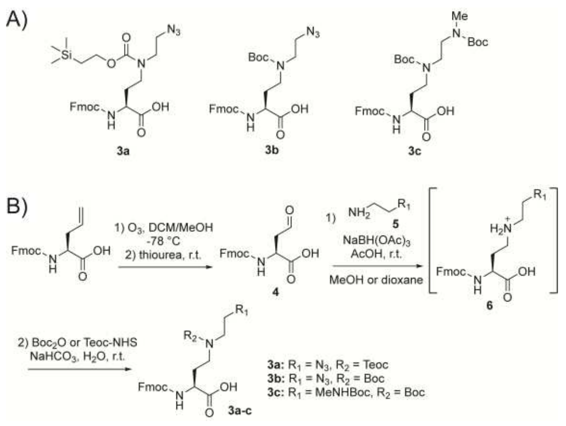

**Figure 7**. (A) Structures of protected residues used in the synthesis of peptides in this study. (B) Synthesis of compounds **3a-c**.

The ability of compound **3a** with its Teoc-protecting group to limit methyl amine formation was first assessed using the peptide XIA (X = residue **1**). **Figure 8A** shows chromatograms of crude peptide prepared using Teoc-protected **3a** versus Boc-protected **3b**. Gratifyingly, no methylamine byproduct was detected when compound **3a** was employed in the synthesis. The same was observed for the longer peptide YGGASVXA, which contains a t-butyl protected serine as well as Teoc-protected residue **1**, **Figure 8B**. This is an interesting case in that t-butyl cation is still generated during the cleavage of this peptide and yet no appreciable methylamine formation is observed. Although we are unsure why the Teoc group seems to protect its neighboring azide from reactive intermediates generated distally in sequence space, this effect seems to be general (*vide infra*). When 7 copies of residue **1** were incorporated in the peptide VXVXVXVXVpPTXVEVXVXV using Teoc-protected **3a**, nearly all of the methylamine-containing byproducts were eliminated. As described before, when the same peptide is prepared using Boc-protected **3b**, multiple methylamine-containing byproducts are observed, with the mono-methylamine byproduct accounting for ~30 % of the crude material, **Figures 3B** and **8C**. Lastly, we investigated the versatility of using compound **3a** under different coupling conditions normally encountered in SPPS employing the model peptide FSXV, where X = residue **1**. **Figure 8D** shows chromatograms for crude peptide prepared using Oxyma/DIC, PyBOP/DIPEA, HOAt/DIC and HATU/DIPEA coupling conditions. The data demonstrate that Teoc-protected **3a** is compatible with all the coupling agents tested and that no byproduct formation was observed, even with having a t-butyl ether protected serine adjacent to residue **1**. In contrast, synthesizing the same peptide employing Boc-protected **3b** using the same suite of coupling agents afforded crude materials that all contained the methylamine byproduct FS$^{Me}$XV, **Figure 8E**.

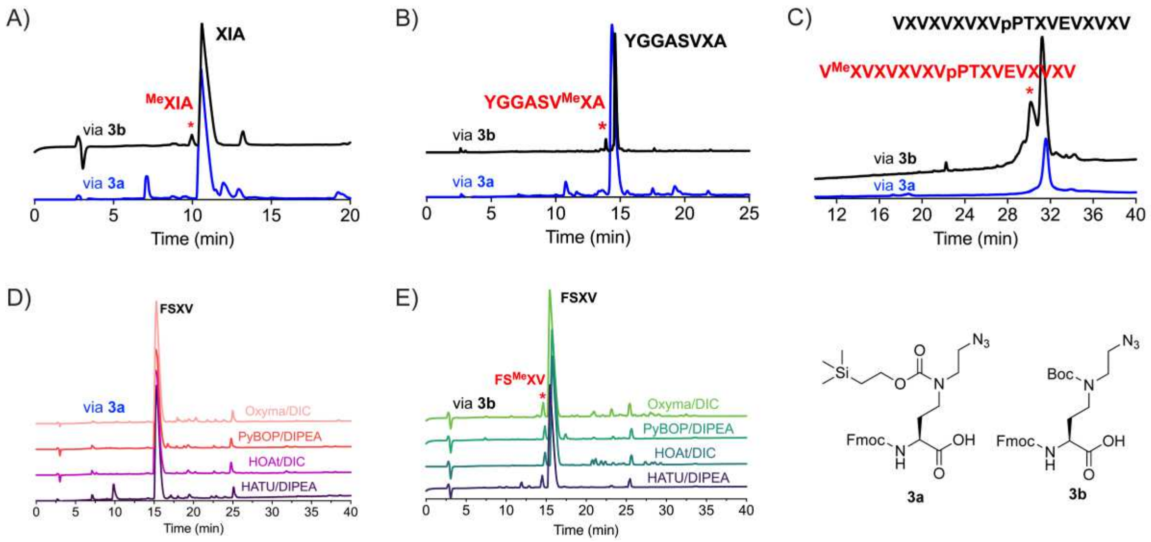

**Figure 8**. Chromatograms of crude peptide prepared using Teoc-protected residue **3a** versus Boc-protected **3b** for peptides: (A) XIA; (B) YGGASVXA; and (C) VXVXVXVXVpPTXVEVXVXV. (D) Chromatograms of crude peptide FSXV synthesized employing Teoc-protected compound **3a** as a function of different coupling reagents. (E) Chromatograms of crude peptide FSXV synthesized employing Boc-protected compound **3b** as a function of different coupling reagents.
X = residue **1** for all peptides. All peptides contain free amino N- and amidated C-termini.

### Conclusion

TFA-mediated cleavage and side chain deprotection of azide-containing peptides produce a byproduct. Byproduct formation can become significant (~ 30%) when multiple copies of azido-containing residues comprise the peptide. NMR and HRMS analysis combined with chemical synthesis demonstrate that the byproduct is consistent with the conversion of azide to methylamine which occurs during peptide cleavage. Data supports a mechanism involving the nucleophilic azide attack of t-butyl cations generated during side chain deprotection of Boc- and t-butyl ether groups. Methyl migration and loss of molecular nitrogen in a Schmidt rearrangement affords an iminium ion whose hydrolysis affords methyl amine. Altering cleavage conditions and scavenger identity was not affective at suppressing this side reaction. However, installing azide-containing residues using the newly reported Teoc-protected compound **3a** proved effective at significantly thwarting the formation of azide-derived methylamine.

### Acknowledgements

This work was supported by the National Cancer Institute, National Institutes of Health (1ZIABC011313).

### Declarations

**Competing Interests**: The authors declare no competing interests.

## Supplementary Files

This is a list of supplementary files associated with this preprint. Click to download.

- 2.SupportingInformationfinal.docx
